# Platelet-rich plasma for androgenetic alopecia: A review of the literature and proposed treatment protocol^☆^^☆☆^

**DOI:** 10.1016/j.ijwd.2018.08.004

**Published:** 2018-09-21

**Authors:** J. Stevens, S. Khetarpal

**Affiliations:** a Northeast Ohio Medical University, Rootstown, Ohio; b Department of Dermatology, Cleveland Clinic Foundation, Cleveland, Ohio

**Keywords:** androgenetic, alopecia, platelet, rich, plasma

## Abstract

Androgenetic alopecia (AGA) is a common hair loss disorder caused by genetic and hormonal factors that are characterized by androgen-related progressive thinning of scalp hair in a defined pattern. By the age of 60 years, 45% of men and 35% of women develop AGA. Currently, U.S. Food and Drug Administration-approved treatments for AGA include oral finasteride and topical minoxidil. Due to the limited number of effective therapies for AGA, platelet-rich plasma (PRP) has become an effective alternative treatment. PRP is an autologous concentration of platelets in plasma with numerous growth factors that contribute to hair regeneration. The growth factors contained within the alpha granules of platelets act on stem cells in the bulge area of the hair follicles and stimulate the development of new follicles along with neovascularization. PRP has become a promising treatment modality for AGA. Although there have been several studies previously reported, a standard practice for PRP preparation and administration as well as a method to evaluate results have not been established. This literature review was conducted to evaluate the effectiveness of PRP for AGA and discuss the various treatment protocols that have been proposed.

## Introduction

Androgenetic alopecia (AGA), also known as female pattern hair loss, is the most common cause of hair loss, affects up to 50% of women over the course of their lifetime, and is known to advance with age and menopause (Rogers and Avram, 2008). AGA is a nonscarring diffuse alopecia due to hormonal and genetic influences and is characterized by the progressive miniaturization of hair follicles, with the transformation of terminal hair into vellus hair (Dinh and Sinclair, 2007). AGA is believed to be attributed to genetic and environmental factors and is known to cause upregulated perifollicular 5-alpha reductase (Rogers and Avram, 2008). The 5-alpha reductase converts testosterone to dihydrotestosterone, which binds to androgen receptors and leads to the activation of genes that transform healthy terminal follicles into vellus-like hairs in androgen-dependent areas of the scalp. These areas are the central, frontal, and parietal regions.

Hair loss can cause considerable psychological and emotional distress and decreased quality of life. Therefore, developing a safe and effective treatment modality can greatly benefit patients in a dermatology practice setting. Current treatment options include minoxidil, finasteride, spironolactone, nutritional supplementation, low-level light therapy, and hair transplantation surgery. Although topical minoxidil and oral finasteride are approved by the U.S. Food and Drug Administration, they have drawbacks including limited degree of clinical improvement in some patients. The efficacy of new hair growth with current U.S. Food and Drug Administration-approved therapies can be unsatisfactory and significant improvement is not always observed.

Platelet-rich plasma (PRP) is a treatment modality that has gained popularity for AGA due to its autologous nature, minimal invasiveness, absence of major side effects, and more affordable cost compared with hair restoration surgery. PRP is an autologous preparation of platelets in concentrated plasma (usually > 1,000,000 platelets/μL or 2-7 times the native concentration of whole blood) (Marx, 2001). Due to its autologous origin and minimally invasive collection technique, the risk of infection and immune rejection is minimized.

## Platelet-rich plasma mechanism of action

Activated platelets are understood to release numerous growth factors and cytokines from their alpha granules as part of the wound healing process. Platelets in PRP become activated when injected into the scalp and release multiple growth factors, which promote hair growth. These growth factors play a role in fibroblast activation, collagen synthesis, stimulation of the extracellular matrix, and overexpression of endogenous growth factors.

Growth factors including platelet-derived growth factor (PDGF), transforming growth factor beta (TGF-β), vascular endothelial growth factor (VEGF), epidermal growth factor (EGF), and insulin-like growth factor-1 (IGF-1) are released by activated platelets in PRP (Alves and Grimalt, 2016) and believed to promote cell proliferation, differentiation, angiogenesis, and chemotaxis that is necessary for new hair regrowth (Kim et al., 2011, Uebel et al., 2006). IGF-1 has been shown to induce and prolong the anagen phase of the hair growth cycle. Platelets also contain dense granules that contain bioactive factors to increase membrane permeability and modulate inflammation (Leo et al., 2015). Dense granules contain serotonin, histamine, dopamine, calcium, and adenosine that increase membrane permeability and modulate inflammation (Asadi et al., 2014, Leo et al., 2015).

The precise mechanism of action of PRP in the promotion of hair growth is still not completely defined. Li et al. (2012b) studied the effects of PRP on hair growth using in vivo and in vitro models. Activated PRP has been reported to induce the proliferation of dermal papilla (DP) cells by activating extracellular signal-related kinase (ERK) and protein kinase B (Akt, an anti-apoptotic signaling molecule) signaling (Alves and Grimalt, 2018, Cervelli et al., 2014, Li et al., 2012b. EGF and PDGF in PRP upregulate the ERK pathway, leading to the increased transcription of genes involved in cellular proliferation and differentiation. In addition, the increased expression of B-cell lymphoma-2 (an anti-apoptotic protein) has been observed in in vitro human DP cells cultured with PRP (Li et al., 2012b). Thus, activated PRP is believed to affect hair cycling by prolonging the length of the anagen phase and preventing apoptosis and the catagen phase. A faster telogen-to-anagen transition was also seen compared with control mice (Li et al., 2012b).

Angiogenesis and increased vascularization of the follicle are thought to be critical for the initiation of the anagen phase (Mecklenburg et al., 2000). Conversely, reduced blood flow and oxygen pressure have been observed in AGA (Goldman et al., 1996). The growth factors in PRP act on stem cells found in the bulge area of follicles, resulting in neovascularization and folliculogenesis (Li et al., 2012a, Li et al., 2012b, Takikawa et al., 2011). Increased β-catenin expression, which is believed to increase DP cell proliferation, survival, and angiogenesis was also observed (Li et al., 2012b). In summary, PRP stimulates hair growth by improving follicle vascularization, inhibiting apoptosis and thereby prolonging the anagen phase, and inducing a faster transition from the telogen to the anagen phase in DP cells.

## Platelet-rich plasma classification systems

Multiple authors have suggested classification systems to promote the standardization of PRP to facilitate interpretation and comparison of different studies and its efficacy. Currently, PRP is an umbrella term that does not distinguish between the various characteristics of what is being used, including platelet concentration, presence or absence of granulocytes, and other factors that can affect hair growth. Mishra’s PRP classification system was based on the presence or absence of white blood cells, activation status, and platelet concentration coefficient compared with whole-blood baseline (Mishra et al., 2012). This classification system was based on available PRP systems in 2006, which included buffy coat and single-spin suspension. Since its publication, technology and knowledge of important PRP attributes have evolved. The double-spin method was developed and produces a higher platelet concentration (> 5 times whole-blood platelet concentration) with little to no red blood cells and neutrophils (Mautner et al., 2015).

In 2009, Dohan Ehrenfest et al. published a PRP classification based on the presence of individual components (platelet and leukocyte concentrations) and the presence or absence of fibrin (Dohan Ehrenfest et al., 2009). Commercially available systems were placed into four main families: pure PRP (P-PRP), leukocyte and PRP (L-PRP), P-PRF (pure platelet-rich fibrin), and L-PRF (leukocyte and platelet-rich fibrin). Pure P-PRP or leukocyte-poor PRP includes preparations without leukocytes and with a low-density fibrin network after activation; L-PRP includes preparations with leukocytes and with a low-density fibrin network after activation; P-PRF or leukocyte-poor platelet-rich fibrin encompasses preparations without leukocytes and with a high-density fibrin network; L-PRF products are preparations with leukocytes and with a high-density fibrin network.

The previous PRP classifications did not address red blood cell content, volume of PRP preparation, or dose of injected platelets in the final volume. In 2016, Magalon et al. proposed a standardized dose/efficiency/purity/activation classification of PRP called DEPA, which is based on four components: dose of injected platelets, efficiency of the production, purity of PRP obtained, and activation process (Magalon et al., 2016). The dose of injected platelets is calculated by multiplying the platelet concentration in PRP by the obtained volume of PRP. The injected dose of platelets should be measured in billions or millions of platelets. The efficiency of the production corresponds to the percentage of platelets recovered in the PRP from blood samples. The purity of the PRP refers to the relative composition of platelets, leukocytes, and red blood cells. The activation process of the classification applies if exogenous platelet activators such as thrombin, calcium gluconate, or calcium chloride were added (Magalon et al., 2016).

## Preparation of platelet-rich plasma

There is still no standardized method of preparation and application of PRP. PRP is produced through cell separation by centrifugation and then injected into androgen-dependent areas of the scalp. Multiple methods of preparation of PRP have been reported in the literature including commercial kits and manual methods using a laboratory centrifuge. Some devices include an accessory to reduce leukocyte count and increase platelet purity. Most preparations use either closed or semi-closed systems, which differ in their ability to concentrate platelets (Dhurat and Sukesh, 2014). This results in suspensions that contain different concentrations of platelets and leukocytes.

All PRP preparation protocols follow a generic method. Blood is collected with an anticoagulant such as citrate (a calcium chelator) to prevent spontaneous blood clotting and consequent platelet activation. Subsequently, whole blood is centrifuged to separate red blood cells, followed by centrifugation to concentrate platelets (Dhurat and Sukesh, 2014). Many protocols include the addition of exogenous platelet activators such as thrombin or calcium chloride prior to administration, which results in an immediate dose-dependent release of growth factors. However, there is no consensus about whether this improves efficacy (Dhurat and Sukesh, 2014). Nonactivated or resting PRP may be injected and spontaneous platelet activation occurs due to exposure to dermal collagen and thrombin (Cavallo et al., 2016). Active growth factor secretion begins within 10 minutes of activation, and a release of > 95% of growth factors occurs within 1 hour ((Marx, 2001). Platelet growth factor synthesis continues for 7 days (Senzel et al., 2009).

PRP is injected sub- or intradermally into the affected scalp regions. The optimal number of treatments and time spaced between them has not been established. The wide variation in reported protocols to obtain PRP may lead to samples with different compositions of platelets, leukocytes, erythrocytes, and growth factor concentrations that may induce different biological responses (Dohan Ehrenfest et al., 2009, Mazzocca et al., 2012). Establishing the significance of these elements is crucial to identify the most effective preparation for AGA.

Platelet concentration factor is the most frequently described parameter and thought to primarily influence PRP efficacy (Magalon et al., 2016). Several studies have discussed the importance of the platelet concentration factor in the promotion of tissue regeneration, which indicates that a concentration two to six times higher than basal platelet count is required for optimal outcomes (Weibrich et al., 2004). A study by Giusti et al. (2009) showed that the optimal platelet concentration for the induction of angiogenesis in human endothelial cells was 1.5 million platelets per microliter. In contrast, higher concentrations of platelets were suggested to decrease the angiogenic potential (Giusti et al., 2009).

In a study by Gentile et al. (2015), a mean of 1.48 million platelets per microliter was shown to stimulate follicular and perifollicular angiogenesis, which is important for active hair growth (Gentile et al., 2015, Uebel et al., 2006). Currently, there is debate in the literature as to whether leukocytes in PRP have a positive or negative effect in AGA. Advocates of PRP high in leukocyte concentration believe the presence of leukocytes provide protection from infection, increase growth factor release, and contribute to angiogenesis, matrix production, and hypercellularity (Dohan Ehrenfest et al., 2009, Dohan Ehrenfest et al., 2012). However, other authors do not recommend the presence of leukocytes in PRP. Neutrophils may release reactive oxygen species and proinflammatory cytokines such as tumor necrosis factor alpha, which may increase inflammation and destroy surrounding tissue (Dohan Ehrenfest et al., 2012, Zhou et al., 2015).

Leukocytes may also increase matrix metalloproteinase levels, which may cause matrix degradation. A recent study assessed the ability of leukocytes in PRP to affect the in vitro biological response on fibroblasts and endothelial cells, and found that they do not rely on the presence of leukocytes (Giusti et al., 2018). Erythrocytes are removed in most PRP preparation methods, but the extent of removal is infrequently reported. These may represent a source of released reactive oxygen species, and act as an inflammatory stress inducer (Magalon et al., 2016). In summary, platelet enrichment, leukocyte, and erythrocyte content may affect key parameters of PRP.

## Discussion

Multiple successful studies have been reported and demonstrate PRP efficacy as a treatment modality for androgenetic alopecia. These studies were selected because they were the most recent articles using PRP for AGA. Dose and administration protocols, evaluation scales, and methods are not standardized, which makes a comparison of the results between the studies difficult. Many studies do not discuss platelet concentration, presence of contaminants such as granulocytes, and injection depth. Other factors to consider are the field effect of PRP in several of the split scalp studies using PRP versus placebo (Table 1).

**Table 1:** Summary of cited studies

Author	Patient characteristics (n, % female)	Objective outcome measures	PRP preparation	Use of activator	Platelet enrichment	Treatment protocol	Follow-up time	Positive results
Alves and Grimalt, 2016	22 (11, 50%)	Hair count, hair density, anagen hair, anagen:telogen ratio, terminal hair density	Single-spin method	Calcium chloride	× 3	3 treatment sessions, 1 month apart	6 months	Yes
Gentile et al., 2015	23 (0, 0%)	Hair density, hair count, epidermal thickness and hair follicle density, number of Ki67 + basal keratinocyte proliferation, number of small blood vessels around hair follicles	Cascade-Selphyl- Esforax system (platelet-rich lipotransfert system)	Ca^2 +^	–	3 treatment sessions, 1 month apart	24 months	Yes
Cervelli et al., 2014	10 (0, 0%)	Hair count, terminal hair density, number of small vessels around follicles, number of basal keratinocytes	Cascade-Selphyl-Esforax	Ca^2 +^	–	3 treatment sessions, 1 month apart	12 months	Yes
Schiavone et al., 2014	64 (0, 0%)	Hair count, hair thickness	GPS III platelet separation system, single spin at baseline and double spin at 3 months	No	× 6-×7	2 treatment sessions, 3 months apart	6 months	Yes
Singhal et al., 2015	10 (2, 20%)	Hair count (hair pull test)	Double-spin method	Calcium chloride	–	4 treatment sessions, 2 weeks apart	3 months	Yes
Gkini et al., 2014	20 (2, 10%)	Hair density	Single spin method (Regenlab SA)	Calcium gluconate in a 1:9 ratio (0.1 ml per 0.9 ml of PRP)	× 5.8	3 treatment sessions, 3 weeks apart followed by booster at 6 months	12 months	Yes
Takikawa et al., 2011	26 (10, 38%)	Hair count, hair diameter	Manual double spin	No	× 6	5 treatment sessions at weeks 0, 2, 4, 6, and 9	4 months	Yes
Puig et al., 2016	26 (26, 100%)	Hair count, hair mass index	Angel PRP system	No	× 2.75-3.4	1 treatment	26 weeks	No
Ayatollahi et al., 2017	13 (0, 0%)	Hair density, hair diameter	Single-spin method (Regenlab PRP Kit-RegenACR)	No	–	5 treatment sessions, 2 weeks apart	3 months	No

PRP, platelet-rich plasma.

Alves and Grimalt (2016) performed a randomized, blinded, half-head study of 25 patients with AGA. Patients had no prior use of medication and were Stages II to V of Hamilton-Norwood and Stages I to III of Ludwig. Each patient received three treatments of PRP, spaced 1 month apart, and were evaluated using phototrichogram and global photography. At 6 months, a statistically significant improvement was seen in mean anagen hairs, telogen hairs, hair density, and terminal hair density in PRP-treated areas compared with baseline. However, increased hair density was the only statistically significant value that increased when compared with the control group. The researchers proposed an application of three initial treatments spaced 1 month apart, then waiting 6 months and performing another three cycles of PRP, with maintenance every 6 months or three cycles of PRP per year (Alves and Grimalt, 2016).

Gentile et al. (2015) conducted a randomized, evaluator-blinded, placebo-controlled, half-head group study of 20 male patients with male pattern hair loss stages II to IV of the Norwood-Hamilton classification. After three treatments at 30-day intervals, patients showed an improvement in mean hair count and total hair density (by computerized trichogram) compared with those who received placebo. A microscopic evaluation and immunohistochemistry were performed 2 weeks after completion of the PRP treatment and also showed an increase in Ki67 + epidermal basal keratinocytes, small blood vessels around hair follicles, and hair follicular bulge cells in PRP-treated hair skin (*p* < .05). These results suggest that PRP may increase keratinocyte proliferation and perifollicular angiogenesis (Gentile et al., 2015).

Cervelli et al. (2014) selected 10 patients with male androgenetic alopecia who were not on any other medication for the previous 12 months. The evaluation was randomized, blinded, with a placebo half-head group, and evaluated by TrichoScan. After 3 months, a mean increase of 27.7 hairs/cm^2^ was found in the treated areas compared with a decrease of three hairs/cm^2^ in the control areas. In addition, terminal hair density improved by 27 ± 15.3 in the treated areas but decreased by 2.1 ± 12.4 in the control area of the scalp (Cervelli et al., 2014).

Schiavone et al*.* (2014) studied 64 patients with AGA, and injected L-PRP and plasmatic proteins in two interventions separated by a 3-month interval. An evaluation was conducted by two evaluators using the Jaeschke scale and with clinical photographs. An improvement by global physician assessment score was observed in all patients by one evaluator, and in 62 of 64 patients by the other (Schiavone et al., 2014).

Singhal et al*.* (2015) treated 10 patients with AGA (Ludwig score I-II, Hamilton–Norwood score 1-4), and administered PRP alone for 3 months, using a control group that was treated with a medical treatment. The sessions of PRP were administered every 2 to 3 weeks for 3 months. An evaluation by pull test showed significant results, and an improvement of hair count, hair thickness, and degree of alopecia was reported (Singhal et al*.,* 2015).

Gkini et al. (2014) studied 20 patients in a prospective cohort study. Patients were treated with PRP for three sessions at 3-week intervals and one treatment at 6 months. The results showed that hair density increased throughout the study with the greatest hair density occurring at three months. Hair density decreased at 6 and 12 months post-treatment but remained above baseline. The researchers proposed a booster treatment at 6 months to maintain and improve the results achieved. Of note, patients with less severe alopecia (Norwood Hamilton Grade II-III) had more favorable results compared with those with more advanced forms of alopecia (Gkini et al., 2014).

Takikawa et al. (2011) studied the use of dalteparin and protamine (D/P) microparticles added to PRP compared with PRP alone. D/P microparticles act as a scaffold to slow the release of growth factors into the injected site. Both the PRP-D/P and PRP treatment groups showed an increased hair count when compared with the control group. The PRP-D/P treatment group showed an increased hair diameter when compared with those who received PRP treatment (Takikawa et al., 2011).

Although there have been many studies supporting the use of PRP in AGA, there have also been studies showing a lack of improvement with PRP. Puig et al. (2016) conducted a double-blind, multicenter, placebo-controlled study of 26 female patients with Ludwig II AGA. After receiving one PRP or normal saline placebo subcutaneous scalp injection, an evaluation was performed using hair count (through photography), hair mass index (Cohen hair check system), and patient opinion surveys at 26 weeks. There was no statistically significant difference in hair mass index or hair count when comparing the PRP-treated and placebo groups. However, 13.3% of PRP-treated patients reported an improvement in ease of styling, hair loss, and hair thickness compared with 0% in the placebo group (Puig et al., 2016).

Ayatollahi et al. (2017) studied 13 male patients with AGA Hamilton grade III to VI. Patients received five injections of 2 to 4 ml PRP by single spin process every 2 weeks. An evaluation was performed at baseline and 3 months after the last injection by standard photographs, trichogram, and hair density and diameter (digital photographic hair analyzer). The results revealed a decrease in anagen, increase in telogen, and decreased anagen/telogen ratio (*p* = .003). No difference in hair count or density was found (Ayatollahi et al., 2017). Given the exact platelet concentration, method of preparation, and presence or absence of granulocytes were unknown, both factors could contribute to the lack of improvement in both studies.

The results from studies conducted to date suggest the promising use of PRP’s therapeutic potential for the treatment of AGA. Seven of 9 studies showed positive results. PRP efficacy was assessed by multiple outcomes: before and after on global photography, hair pull test, mean hair count and hair density, anagen-to-telogen ratio, and patient satisfaction surveys. Norwood-Hamilton and Ludwig scales were used to classify the degree of hair loss. The volume of whole blood drawn, centrifugation settings, and mean platelet enrichment differed between the studies. Treatment protocols ranged from 1 to 6 sessions with time intervals spaced 2 weeks to 3 months. The total volumes injected per session ranged from 2 to 12 cc, and have been reported as 0.05 to 0.6 cc/cm^2^ of the treated scalp.

Evidence of improvement after treatment with PRP was most commonly reported after the third month. Most studies did not follow the patients' clinical course beyond 6 months. However, some patients with a long-term follow-up period showed a decline in hair density. Four of 20 male patients experienced progressive hair loss that was most apparent at 16 months (Gentile et al., 2015). A reduction in hair density at months 6 and 12 after the last treatment was reported by Gkini et al. (2014), but hair density remained above baseline (Kim et al., 2011). The most common side effect reported in the reviewed studies was temporary pain at the injection site. No major adverse effects were reported.

## Proposed treatment protocol

**Fig. 1:**
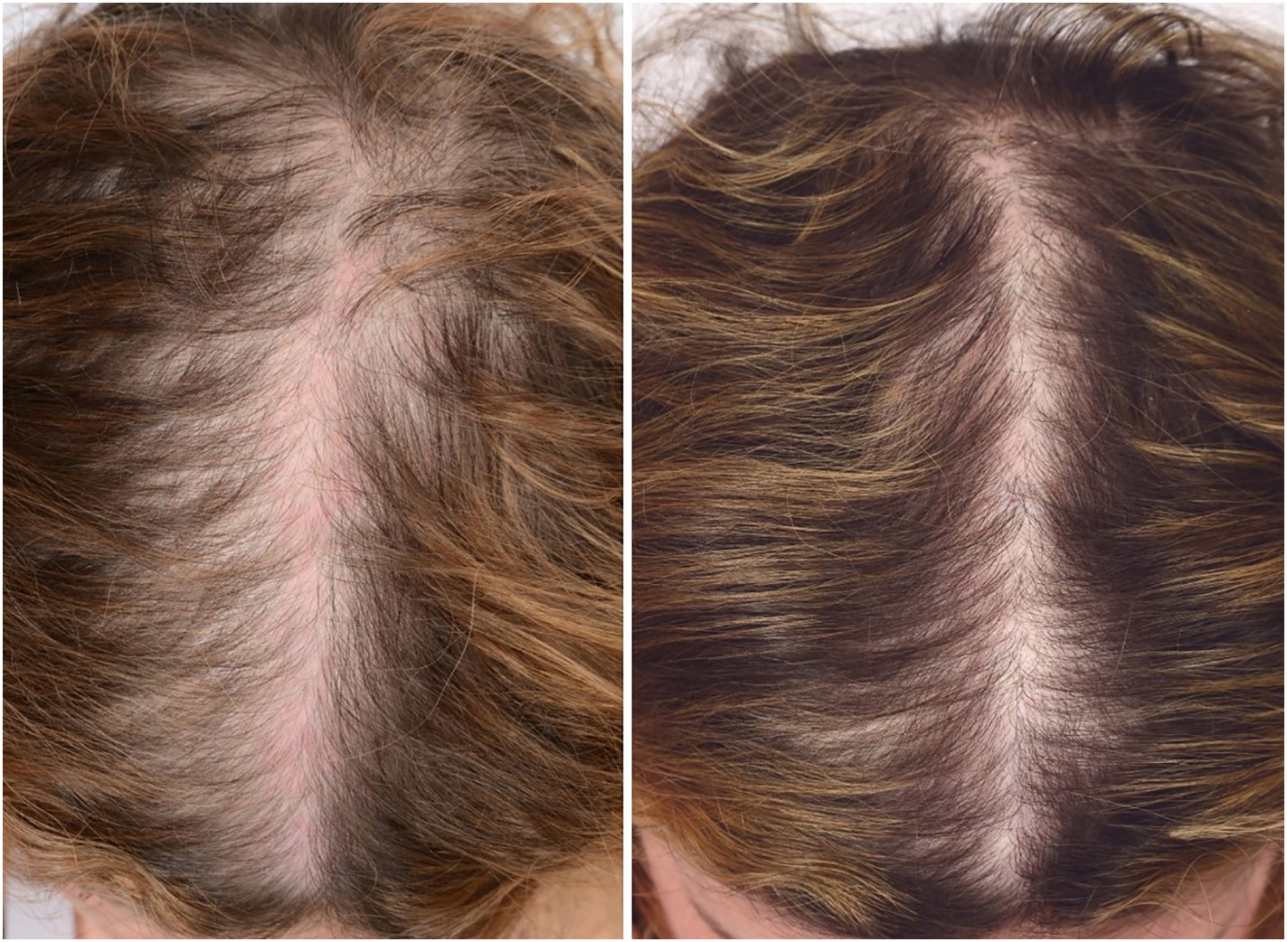
A 46-year-old woman with androgenetic alopecia, before and 3 months after 3 platelet-rich plasma treatment sessions.

**Fig. 2:**
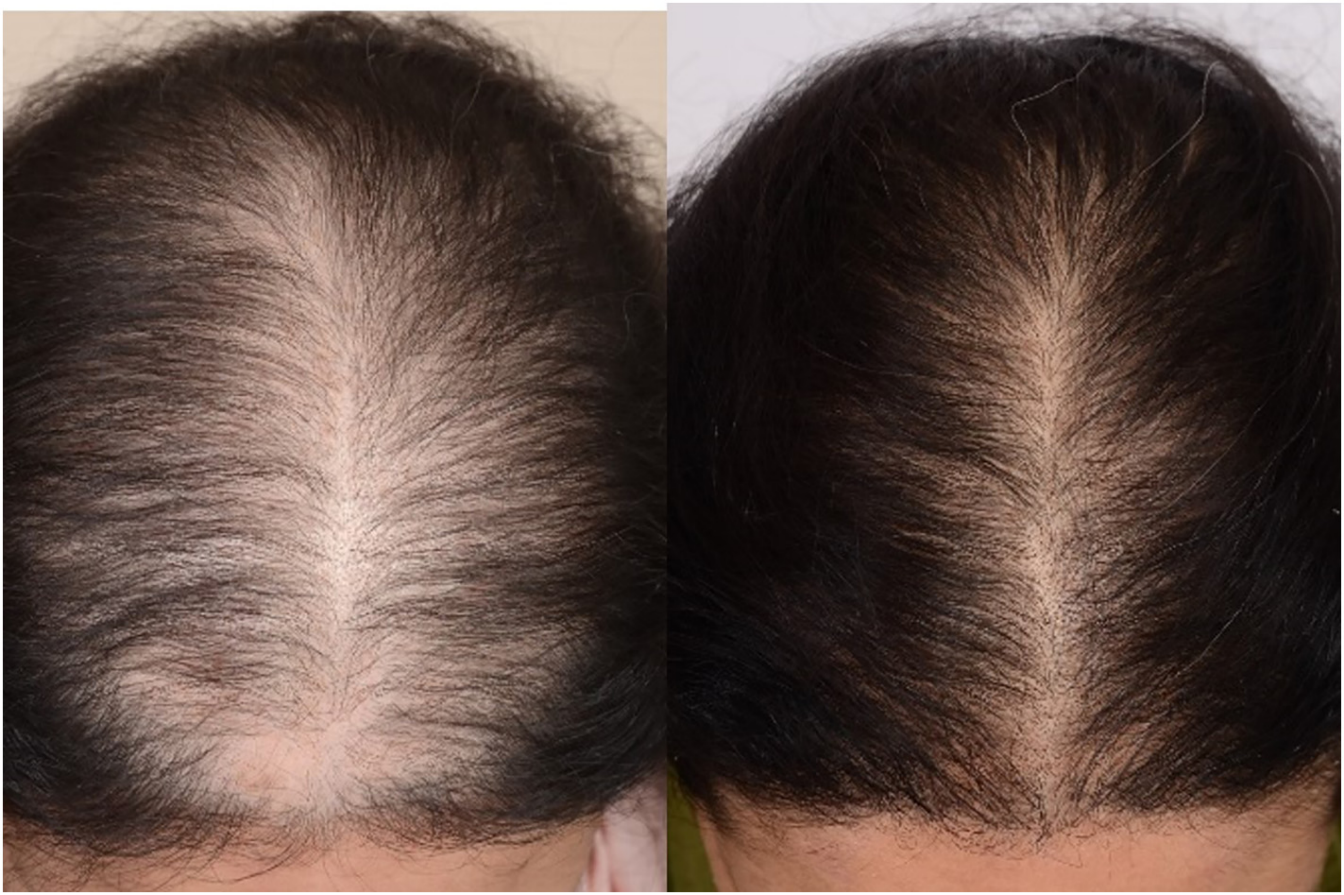
A 31-year-old woman with androgenetic alopecia, before and 4 months after five platelet-rich plasma treatment sessions.

From clinical experience, we recommend PRP as a coadjuvant treatment for AGA, and encourage patients to continue topical and/or oral therapies (e.g., minoxidil, spironolactone, and finasteride) because PRP does not suppress the hormonal component of androgenetic alopecia. Based on our review of the published studies, we suggest PRP preparation by a single spin centrifugation method to produce pure PRP with a platelet enrichment of 3 to 6 times the mean concentration of whole blood and minimize granulocytes. Addition of an activator before administration may be beneficial in growth-factor release because most positive studies included one, such as calcium chloride or calcium gluconate. We recommend the administration of PRP as subdermal depo bolus injections because this is less painful and an overall more efficient injection technique. Subdermal depo bolus injections allow for the diffusion of PRP and result in fewer injections. Injections should be spaced out in the thinning area, which is typically along the hairline, part, vertex, and crown of the scalp. The volume of PRP varied greatly between the positive studies. Treatment intervals should include monthly sessions for the first 3 months, then every 3 months for the first year (6 treatment sessions in first year at months 1, 2, 3, 6, 9, and 12). However, three monthly sessions followed by sessions at 6-month intervals have also been effective. Overall, male and female patients have had positive results from PRP injections in AGA in terms of regrowth, increased hair density, and improved quality of life (Figs. 1 and 2).

## Conclusion

The use of PRP to treat AGA is promising based on the results of the reviewed clinical studies. Safety issues, side effects, and downtime seem to be minimal. Although PRP does appear to be beneficial, the preparation, dosage, number, and interval of treatment sessions, as well as injection technique, vary between the studies due to a lack of standardization of PRP preparation. This makes inferring conclusions about its clinical efficacy difficult. To further classify the effects of PRP on hair regrowth in AGA, randomized placebo-controlled studies with larger sample sizes are needed. Such studies would need to report frequency of injections, concentration of PRP, and include long-term follow up to determine how long-term results are sustained. The optimal PRP preparation for AGA is still unknown, and requires further investigation where PRP variables are reported.
